# The familial risk and heritability of multiple sclerosis and its onset phenotypes: A case–control study

**DOI:** 10.1177/13524585231185258

**Published:** 2023-07-12

**Authors:** Graysen Steele Boles, Jan Hillert, Ryan Ramanujam, Helga Westerlind, Tomas Olsson, Ingrid Kockum, Ali Manouchehrinia

**Affiliations:** Department of Public Health Sciences, Karolinska Institutet, Stockholm, Sweden; Department of Clinical Neuroscience, Karolinska Institute, Stockholm, Sweden; Department of Clinical Neuroscience, Karolinska Institute, Stockholm, Sweden; Clinical Epidemiology Division, Department of Medicine, Solna, Karolinska Institutet, Stockholm, Sweden; Department of Clinical Neuroscience, Karolinska Institute, Stockholm, Sweden; Centre for Molecular Medicine (CMM), Department of Clinical Neurosciences, Karolinska Institute, Stockholm, Sweden; Department of Clinical Neuroscience, Karolinska Institute, Stockholm, Sweden; Centre for Molecular Medicine (CMM), Department of Clinical Neurosciences, Karolinska Institute, Stockholm, Sweden; The Karolinska Neuroimmunology & Multiple Sclerosis Centre, Department of Clinical Neurosciences, Karolinska Institutet, Stockholm, Sweden; Centre for Molecular Medicine (CMM), Department of Clinical Neurosciences, Karolinska Institute, Stockholm, Sweden

**Keywords:** Multiple sclerosis, primary progressive, relapsing onset, familial risk, heritability

## Abstract

**Background:**

The two main phenotypes of multiple sclerosis (MS), primary progressive (PPMS) and relapsing Onset (ROMS), show clinical and demographic differences suggesting possible differential risk mechanisms. Understanding the heritable features of these phenotypes could provide aetiological insight.

**Objectives:**

To evaluate the magnitude of familial components in PPMS and ROMS and to estimate the heritability of disease phenotypes.

**Methods:**

We used data from 25,186 MS patients of Nordic ancestry from the Swedish MS Registry between 1987 and 2019 with known disease phenotype (1593 PPMS and 16,718 ROMS) and 251,881 matched population-based controls and 3,364,646 relatives of cases and controls. Heritability was calculated using threshold–liability models. For familial odds ratios (ORs), logistic regression with robust sandwich estimator was utilized.

**Results:**

The OR of MS diagnosis in those with a first-degree family member with ROMS was 7.00 and 8.06 in those with PPMS. The corresponding ORs for having a second-degree family member with ROMS was 2.16 and 2.18 in PPMS. The additive genetic effect in ROMS was 0.54 and 0.22 in PPMS.

**Conclusion:**

Risk of MS increases by several folds in those with a relative with MS. The likelihood of developing either disease phenotype appears independent of genetic predisposition.

## Introduction

Over 230 genetic variants have been identified which contribute to increased risk of multiple sclerosis (MS),^1^ and the presence of certain environmental factors may further influence the outcome in these individuals.^2^ Our previous population-wide study of MS familial aggregation in Sweden estimates heritability of MS to be between 35% and 75%.^3^ However, familial aggregation and heritability estimates for different MS phenotypes as well as for MS severity remain unknown. Primary progressive MS (PPMS), which presents in a minority of patients, manifests different characteristics than relapsing onset MS (ROMS). These include equal sex proportion (as opposed to 3:1 female–male ratio in ROMS), older age at onset (AAO) and a substantially more aggressive course from the disease onset. PPMS also shows lower levels of inflammation (indicating lack of typical MS relapses) yet more extensive brain lesions and atrophy.^4^ Compared with ROMS, PPMS presents with reduced satisfactory response to disease modifying treatments (DMTs).

Since MS is a relatively rare disease with a prevalence of 189 per 100,000 individuals in Sweden, the probability that multiple relatives within a familial group share the conditional phenotypes without the presences of a strong heritable influence is correspondingly low. Therefore, investigations of familial risk and heritability of MS phenotypes may provide instrumental knowledge into disease risk and might be clinically relevant for patient counselling and differential diagnosis of phenotypes. Comparison of familial risk and heritability of MS phenotypes may further guide future epidemiological and molecular research by providing insights into the disease aetiology.

Our aim in this work is to examine and compare the degree of familial risk and heritability between PPMS and ROMS phenotypes.

## Method and material

### Study population

We conducted a case–control study to estimate the familial risk and heritability of MS in patients with PPMS and ROMS. The subjects included were enumerated on a national level and consisted of all persons with MS in Sweden diagnosed with MS between 1987 and 2019 for whom records were available. Cases of MS were defined as by having at least three International Classification of Diseases (ICD)-10 G35.9 (unspecified MS) or ICD-9 340x (MS) diagnoses in the Swedish national patient register (NPR), or by having been registered in the Swedish MS registry (SMSreg). The development of the NPR began in the 1960s and includes information on diagnosis according to ICD standards, dates, and surgery codes which have been previously described elsewhere.^5^ The SMSreg is a quality registry which began in 2001 and is used specifically to record all diagnosis, clinical courses, and demographic data of MS cases within Sweden, of which approximately 85% are included.^6^

The phenotype of MS for each patient in this study remained the same and was defined by the recorded type at the time of MS diagnosis by the practicing MS specialist neurologist and entered into the SMSreg. For each MS case, 10 sex-, age-, and residential area-matched controls were identified by statistics Sweden. Requirements for controls were that they be alive at the time of MS onset of their respective case (index date), reside in Sweden and have no previous records of an MS diagnosis. The Swedish Cause of Death Register (CDR), a complete recording of all causes of death (by ICD code) in Sweden since 1952,^7^ was used for the identification of deaths in Sweden before 1987 for exclusionary purposes and to ensure that all patients, controls and their relatives were alive at the start of nationwide data collection in NPR in 1986. Hence, any individual who had died before 1987 were excluded from the study, thereby limiting the possibility of misclassification bias. Finally, we utilized the Swedish Multi-Generational Register (MGR) to construct the familial relations. The MGR is a registration which utilizes a majority of individuals living in Sweden (up to 97%) and are listed based on their relationship to an index person (usually parents) born between 1932 and 1 January 1961.^8^

Ethical approval for this project has been obtained from the Stockholm regional ethical committee at Karolinska Institutet (EPN). DNR: 2017/1378-31 and was performed in accordance with the ethical standards set by the 1964 declaration of Helsinki and its later amendments.

### Statistical analysis

For the estimations of familial risks, we used a method described previously.^9^ In short, we calculated the odds of being diagnosed with MS in the family members of those diagnosed with either ROMS or PPMS. We categorized family members by first-degree (parents, offspring and full-siblings) and second-degree relatives (grandparents, grandchildren, uncles/aunts, nieces/nephews and half-siblings). For the calculations of the odds ratios (ORs), we employed conditional logistic regression with robust sandwich estimator which compensates for familial clustering. The OR was separately calculated for PPMS and ROMS for each family relation as well as combined estimates for first- and second-degree relatives. In a sensitivity analysis, we also calculated the ORs for developing either phenotype of MS (ROMS or PPMS) while having a first or second-degree relative with the same phenotype, separately.

Heritability was calculated using threshold–liability models that incorporate the time aspect.^10^ We included first- and second-degree relatives and right-censored individuals at age 85 years to account for the lifetime risk for MS. Heritability of MS onset age was calculated using data from full-sibling and individuals without any first- or second-degree with MS (an unrelated sample). To estimate heritability, a linear mixed effects model was employed, taking into account both fixed effects (i.e. sibling status and sex) and random effects (i.e. familial relatedness). Heritability was then calculated as the proportion of the genetic variance out of the total variance, providing an estimate of the genetic component’s influence on the observed differences in age at disease onset. A 95% confidence interval (CI) for the heritability estimate was calculated using the quantiles of the 1000 bootstrap sample distribution.

All statistical analyses were performed using R version 4.0.

## Results

Demographic data for included individuals stratified by onset phenotype is presented in Table 1. A total of 25,186 MS cases were identified within the Swedish MS registry with available phenotype data, of which 1593 were PPMS and 16,718 were ROMS. Females outnumbered males in both PPMS and ROMS, with 55.2% of cases of PPMS and 71.6% of cases of ROMS, and the age of MS phenotype diagnosis varied with 44 recorded median age (Quartiles of 34 and 51) among PPMS cases and 29 recorded median age (Quartiles of 22 and 38) among ROMS cases.

**Table 1.** Demographic data of individuals with MS and controls (*N* = 277,067).

	Cases with unknown MS phenotype (*N* = 6875)	Primary progressive MS (*N* = 1593)	Relapsing onset MS (*N* = 16,718)	Control (*N* = 251,881)	Total (*N* = 277,067)
Sex					
Female	4529 (65.9%)	879 (55.2%)	11,477 (71.6%)	171,785 (68.2%)	188,670 (68.1%)
Male	2346 (34.1%)	714 (44.8%)	4548 (28.4%)	80,096 (31.8%)	88,397 (31.9%)
Calendar year of birth					
Median	1946	1951	1966	1959	1955
Quartile 1, Quartile 3	1937, 1957	1944, 1959	1955, 1978	1945, 1973	1945, 1966
Age of index					
Number missing	0	0	7	0	7
Median	52	44	29	35	40
Quartile 1, Quartile 3	41, 62	34, 51	22, 38	23, 49	29, 50

MS: multiple sclerosis.

### Familial risk

The ORs for developing MS for each familial group are presented in Table 2. These correspond to the odds of having at least one family member of given degree with MS for each of the phenotypes. In general, there was a significant increased risk of MS among first- and second-degree relatives of patients with either phenotype, while expectedly MS risk decreased substantially among second-degree relatives compared with first-degree relatives.

**Table 2.** Familial reoccurrence risk of MS (odds ratios and 95% confidence intervals) in first- and second- degree relatives of MS cases with relapsing or progressive onset MS.

	Relapsing onset MS (odds ratio (95% CI))	Primary progressive MS (odds ratio (95% CI))
First-degree relatives	7.00 (6.47–7.55)	8.06 (4.74–12.76)
Parent	6.78 (5.85–7.86)	6.54 (4.63–9.16)
Offspring	6.87 (6.14–7.68)	3.56 (1.98–6.08)
Full-sibling	8.65 (7.83–9.57)	9.51 (7.14–12.65)
Second-degree relatives	2.16 (1.98–2.36)	2.18 (1.60–2.91)
Half-sibling	2.40 (1.89–3.00)	1.15 (0.35–2.85)
Grandchildren	2.26 (1.68–2.97)	5.21 (0.75–24.19)
Grandparent	1.25 (0.92–1.67)	0.23 (0.01–1.04)
Aunt/uncle	2.49 (2.09–2.95)	3.46 (2.36–4.94)
Nieces and nephews	2.56 (2.27–2.89)	2.23 (1.06–4.20)

MS: multiple sclerosis; CI: confidence interval.

**Figure 1.**
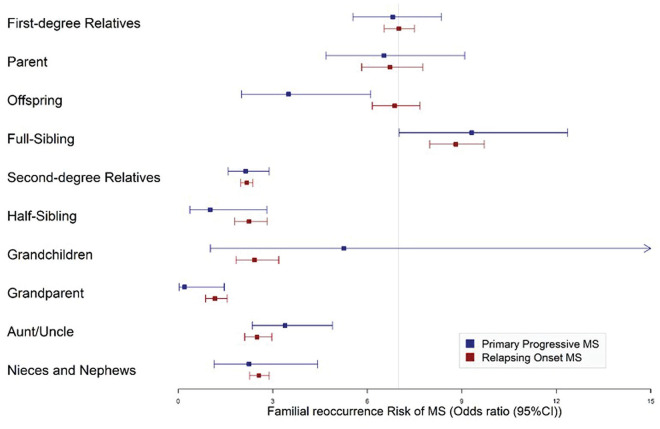
Forest plot of familial re-occurrence risk of MS presented with odds ratios and corresponding 95% confidence intervals in patients with relapsing or progressive onset MS.

The familial risk of MS development for ROMS was 7.00 (95% CI: 6.47–7.55) in first-degree and 2.16 (95% CI: 1.98–2.36) in second-degree relatives. The corresponding estimates for PPMS was 8.06 (95% CI: 4.74–12.76) in first-degree and 2.18 (95% CI: 1.60–2.91) in second-degree relatives. The familial risk of MS for both ROMS and PPMS was the highest among whole siblings, with estimates of 8.65 (95% CI: 7.83–9.57) and 9.51 (95% CI: 7.14–12.65), respectively. Overlapping confidence intervals between PPMS and ROMS for each familial group suggest no significant differences in the familial risk of developing MS between the two phenotypes. This was also the situation among all first- and second-degree relatives, where the confidence intervals in the ORs of developing MS overlapped between the phenotypes. The results are summarized and visualized in Table 2 and Figure 1.

### Degrees of heritability

**Figure 2.**
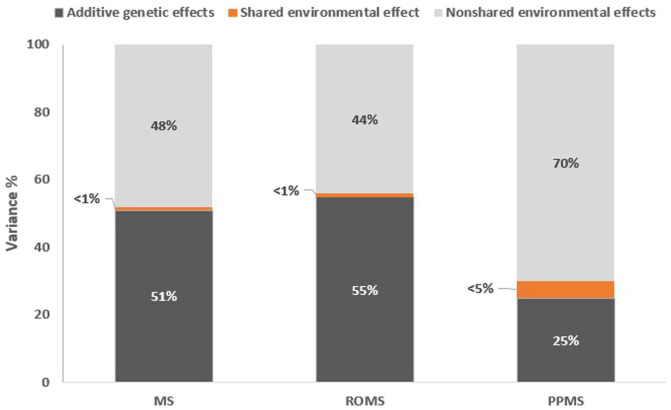
Proportion of variance explained by additive genetic effects, and shared/non-shared environmental effects in MS, ROMS and PPMS.

The heritability of MS, ROMS and PPMS was estimated to be 51% (95% CI: 41‒60), 55% (95% CI 39‒71) and 23% (95% CI: 0–0.97), respectively. This corresponds to the proportion of disease risk explained by additive genetic effects, under the assumption of independent effects. We did not observe any significant contribution from shared environment in overall MS or ROMS (<1%); however, the shared environmental effect in the PPMS group was estimated to be approximately 5% (95% CI: 0–38) (Figure 2).

We analysed data from 16,929 patients with MS with available MS onset age (mean: 34, SD ± 11) including 552 full-siblings (264 sibling pairs and 8 sibling trios). We estimated the heritability of MS onset age to be approximately 21.9% (95% CI: 14.6–29.5). MS onset age was defined as the date of first sign or symptom suggestive of MS recorded by a neurologist.

## Discussion

Here, we have shown evidence that the familial risks between the two MS onset phenotypes for first- and second-degree familial relations are comparable; that is, there is no significant difference in the risk of MS development associated with MS phenotypes. In addition, we found similar estimates of heritability between the phenotypes. Taken together, this indicates that the familial risk of developing MS is consistent between phenotypes, with similar MS risk for relatives of patients with ROMS and PPMS.

Our findings for familial risk reflect similar results as those concluded from the Nielsen et al.,^11^ Song et al.^9,12^ and Westerlind et al.^3^ studies conducted in Denmark and Sweden, where full-siblings showed the highest risk of developing MS followed by parents/offspring, half-siblings and grandparents/grandchildren, respectively. For some categories, case relatives exhibited statistically insignificant or borderline significant *p*-values and wide confidence intervals, which is likely attributable to low power, especially for PPMS cases among grandchildren/grandparents.

Comparable familial risk of MS between different phenotypes of the disease suggests shared aetiology between them and perhaps the demographic and clinical differences seen between ROMS and PPMS are explained by other factors than genetics.

We observed a lower shared genetic component in the heritability of PPMS phenotype as compared with the ROMS, which is most likely due to the small sample size and uncertainty around the estimates. Given that there was no *p-*value utilized to calculate the heritability and the aforementioned lack of subjects for PPMS groups, we cannot rule out the possibility of a difference in heritability between ROMS and PPMS. However, given that results may vary for heritability, it still reinforces the *a priori* knowledge that there is a genetic and environmental interaction in MS, and that the genetic influence is almost equivalent to that of the environmental.

These findings are in line with previous estimates of heritability from a large-scale meta-analysis of twins.^13^ Estimates from familial-based heritability methods are generally higher than those obtained from single nucleotide polymorphism (SNP) panels.^1^ While familial-based heritability estimates are prone to overestimating the heritability, SNP-based estimates may underestimate the genetic component due to a lack of information in sequences not adequately captured or modelled, such as low frequency/rare variants and gene–gene interactions. Hence, the true heritability estimates most likely fall between whole-genome SNP-based and family-based heritability estimates. There is also evidence that the environmental risk may change over time in individuals predisposed to MS,^14^ indicating that heritability may not be static over an individual’s life. A high onset age heritability would provide evidence to the contrary; however, the estimate of 21.9% is consistent with this premise. As the current results represent a decrease from our previous heritability estimates in Sweden,^3^ it is very likely that this gap in ‘missing heritability’ between SNP-based methods and familial-based studies will continue to reduce with the introduction of sequencing-based methods and larger familial-based heritability studies.

One strength of this study is the use of a large, population-based nationwide cohort. The incorporation of SMSreg along with other high-quality Swedish data registries allowed for a more precise analysis of all available cases within the entirety of Sweden. While this study may be generalizable to the Swedish population, it may not extend to other settings where a difference in shared genetic/environmental components may exist less than that of Swedish population. However, our results have similarities with previous studies examining familial risk and heritability of MS, and our heritability estimates of 50% for MS are in line with the results of a meta-analysis of heritability of MS in twins.^13^

Although the sample size derived from the registries was substantial (*n* = 25,186), the number of PPMS patients (*n* = 1593) has a comparatively low power when compared with that of ROMS patients (*n* = 16,718), which created wide confidence intervals for familial risk estimates among half-siblings, grandchildren, grandparents and the heritability estimates. Another limitation may exist within the diagnosis of these two phenotypes, as their phenotypic expressions could be both ambiguous and vary depending on an individual’s subjective interpretations before seeking medical help. This misdiagnosis could lead to an erroneous classification as ROMS or PPMS, depending on when patients seek care, as could the tendencies of the individual neurologist. It is likely that this would have little-to-no bias on our between-group results but could affect the precision of our OR estimates. Nevertheless, our results may point towards the possibility that PPMS, is not, in all actuality, a seperate phenotype of MS, but rather similar to secondary progressive multiple sclerosis (SPMS) – the later stage of ROMS – but without the former diagnosis of ROMS. This assumption would further explain our results of familial risk between the two phenotypes in that the minor differences observed could be explained by misdiagnosis, and therefore, a misclassification of the exposure. Nevertheless, with overlapping confidence intervals, the small differences observed between the phenotypes are already possible by chance.

The generational effect could also present issues, in which individuals could have died before 1987, therefore causing left truncation and not allowing them to be included in the study with a recorded diagnosis within the same time frame as their relatives. Typically, this would happen among older people, which would result in discrepancies among the parent/offspring groups and grandparents/grandchildren groups, and would possibly explain the low power within the PPMS phenotype as it tends to have a late-life diagnosis.

Future research should examine more complex severity traits in lieu of MS phenotypes to determine whether phenotype onset and diagnosis is related to unexplored genetic or environmental risk factors. Further adjustments might include household and environmental factors that could contribute to increased risk of developing either phenotype, especially considering that shared environmental influences during adolescence have been shown to have an effect on MS onset age.^2^

In conclusion, while there is a significant increase in familial risk on the overall outcome of MS, no significant differences were found in familial risk between PPMS and ROMS.
